# *Chlorella vulgaris* Supplementation Attenuates Lead Accumulation, Oxidative Stress, and Memory Impairment in Rats

**DOI:** 10.3390/toxics13040313

**Published:** 2025-04-18

**Authors:** Juan Pablo Diaz, Eduardo Pena, Samia El Alam, Cecilia Matte, Isaac Cortés, Leonardo Figueroa, Patricia Siques, Julio Brito

**Affiliations:** 1 Faculty of Natural and Renewable Resources, Arturo Prat University, Iquique 1100000, Chile; jpdiaz@unap.cl; 2 Núcleo de Investigación Aplicada e Innovación en Ciencias Biológicas, Facultad de Recursos Naturales Renovables, Universidad Arturo Prat, Iquique 1110939, Chile; 3 High Altitude Medicine Research Center, Arturo Prat University, Iquique 1100000, Chile; selalam@unap.cl (S.E.A.); mattececilia@gmail.com (C.M.); patricia.siques.lee@gmail.com (P.S.); julio.brito28001@gmail.com (J.B.); 4 Mathematic Department, Engineer Faculty, Atacama University, Copiapó 1530000, Chile; isaac.cortes@uda.cl; 5 Chemical Department, Science Faculty, University of Tarapaca, Arica 1000000, Chile; ifigero.arica@gmail.com

**Keywords:** lead, *Chlorella vulgaris*, oxidative stress, memory, supplementation

## Abstract

Lead is a harmful heavy metal known to alter the environment and affect human health. Several industries have contributed to the increase in lead contamination, making it a major global concern. Thus, remediation strategies are necessary to prevent lead bioaccumulation and deleterious health effects. The aim of this study was to determine the capacity of the green microalga *Chlorella vulgaris* (*C. vulgaris* or CV) to remove lead in an animal model and prevent the accumulation of this heavy metal in the principal organs (brain, liver, and kidney) and blood. Forty male Wistar rats were randomly assigned to four groups (n = 10): control group (CT); *C. vulgaris* supplementation group, 5% of the diet (CV); lead acetate administration group, 500 ppm (Pb); and *C. vulgaris* supplementation group, 5% of the diet plus lead acetate administration group, 500 ppm (CV–Pb). After 4 weeks of exposure, we measured lead accumulation, memory function, oxidative stress, and antioxidant activity (SOD and GSH). Lead exposure altered memory function, increased oxidative stress in the brain and kidney, and increased SOD activity in the brain. Supplementation with *C. vulgaris* restored memory function to control levels; reduced oxidative stress in the brain and kidney; and decreased the accumulation of lead in the liver, kidney, and blood of rats exposed to lead. Based on our results, *C. vulgaris* is a lead chelating and antioxidant agent in animal models.

## 1. Introduction

Lead is a harmful heavy metal and an environmental pollutant; human activities such as mining, smelting, lead battery production, and the crystal and ceramic industries are the main sources of lead. This toxic metal can seriously affect organisms and can cause acute (cough, headache, confusion, abdominal pain, hemolytic anemia, and hepatic and pancreatic inflammation, among other symptoms) and chronic or severe intoxication (peripheral neuropathy, anemia, colicky abdominal pain, impaired kidney function, and cardiac dysrhythmia) [1,2,3]. Moreover, lead affects the kidneys, liver, hematopoietic system, cardiovascular system, reproductive system, and peripheral and central nervous systems [2,4,5]. With respect to the central nervous system, several studies have demonstrated that lead exposure causes cognitive impairment, alterations in executive function, abnormal social behavior, and fine motor control perturbations in humans [6,7,8].

One of the main effects of lead in cells is the inducement of oxidative stress, which causes tissue injury and impaired liver, kidney, and brain function [4,9]. Oxidative stress is a condition produced by an excess level of reactive oxygen species (ROS) that are not scavenged by the antioxidant system. Additionally, oxidative stress is related to several cardiovascular and neural pathologies [10,11,12]. Studies have demonstrated that workers exposed to lead have increased levels of lipid peroxidation that generate oxidative products (mainly malondialdehyde, MDA) and decreased total antioxidant capacity in the bloodstream [13,14]. Interestingly, the main antioxidant, superoxide dismutase (SOD), which is the first line of defense against ROS, has also been studied in workers exposed to different levels of lead, and it has been shown that SOD activity is greater in workers with low and medium levels of exposure to lead [15]. Moreover, another study showed that SOD activity was increased in children with neurological disorders and higher blood lead levels [16].

There are several traditional chemical chelating agents for the treatment of lead toxicity; however, their efficiency is limited by their own toxicity and side effects [17,18]. A recent study proposed microalgae as a potent antioxidant and chelator for toxic heavy metals [19]. One such microalga is *Chlorella vulgaris (C. vulgaris)*, which is a green algae that has several health benefits when used as a dietary supplement, decreasing the risk factors for several pathologies such as nonalcoholic fatty liver disease [20], cardiovascular pathologies [21], and metabolic alterations [22], as well as improving working memory [23]. The latter effect is relevant as lead can generate critical alterations in the cognitive system through oxidative stress [24], an effect that has also been described for other heavy metals such as cadmium and arsenic [25,26]. Although physiological levels of ROS are necessary for synaptic plasticity and cognitive function [27], studies have shown that ROS are typically neurotoxic molecules that produce detrimental effects through the oxidation of essential macromolecules such as enzymes and cytoskeletal proteins. In addition, excess levels of ROS in some cases are associated with decreased cognitive function [11,28]. Therefore, the aim of this study was to evaluate the ability of *C. vulgaris* supplementation to decrease lead accumulation in rats and thus reduce the oxidative stress and cognitive alterations induced by heavy metal exposure.

## 2. Materials and Methods

### 2.1. Animal Care

Animals were obtained from the vivarium of the High Altitude Medicine Research Center, Arturo Prat University, Iquique, Chile. All procedures and protocols were carried out with the approval of the Research Ethics Committee of Tarapacá University. Furthermore, the study was carried out in accordance with the ethical standards for the management of experimental animals (Chilean Law 20,380, Art 7, 3 October 2009). The young (2-month-old) male Wistar rats were housed in cages (3 rats/cage) under 12 h of light and 12 h of darkness at a temperature of 22 ± 2 °C. Food (22.0% crude protein, 5.0% crude fiber, 9.0% ash, and 12% moisture; 5 POO^®^, LabDiet, Prolab RMH3000, St. Louis, MO, USA) and water were provided ad libitum. When the rats reached 5 months of age, they were separated into individual cages until they reached 6 months of age.

### 2.2. Study Protocol

Forty male Wistar rats (6 months old) were used in this study. The rats were housed in individual cages at a temperature of 22 ± 2 °C with a light cycle of 12 h of light and 12 h of darkness. The feed was provided at 30 g/day and contained 22.0% crude protein, 5.0% crude fiber, 9.0% ash, and 12% moisture (5 POO^®^, LabDiet, Prolab RMH3000, St. Louis, MO, USA), and water was provided ad libitum. No physical exercise was performed; however, movement within the cage was not restricted. Weight was measured using an AccuLab V-1200^®^ electronic balance (Marrero, LA, USA).

The rats were randomly distributed into 4 experimental groups: the control (CT; n = 10) group, the *C. vulgaris* (CV; n = 10) group, the lead (Pb; n = 10) group, and the *C. vulgaris* plus lead (CV–Pb; n = 10) group. Lead was continually administered as 500 ppm lead acetate (Sigma–Aldrich 316512, Burlington, MA, USA) through the drinking water, and *C. vulgaris* (FEBICO, Far East Bio Tec. Co., Ltd., Taiwan) was provided as 5% of the diet. The reference values for the chemical characterization of *C. vulgaris* are 9.43% moisture, 50.2% crude protein, 41.9% carbohydrates, 3.22% crude lipids, and 4.66% ash [29]. The protocol period was 30 d. Body weight (BW; g) was measured with an electronic balance (AccuLab V-1200^®^, Lake Country, IL, USA). At the end of the study period, the rats were anesthetized with ketamine (90 mg/kg BW) and euthanized through fatal thoracotomy [30]. Organ (liver, kidney, heart, and brain) and blood samples were then taken and immediately stored at −80 °C.

### 2.3. T-Maze

To assess the spatial memory of the rats, a T-maze with dimensions similar to those described by Deacon and Rawlins [31] was used. The rats started at the base of the T-maze and could choose one of the arms spontaneously (right or left). An entry was recorded when the animal placed all four paws inside one arm. Nine trials were performed at 30 s intervals [32].

### 2.4. Lipid Peroxidation

Malondialdehyde (MDA) concentrations (µmol/L) in liver, kidney, brain, and plasma samples were measured using a colorimetric assay. First, 30 mg of tissue was homogenized in 300 µL of RIPA buffer (50 mM Tris-HCl, 1% Triton X-100, 150 mM NaCl, and 0.1% SDS) for 2 min in a homogenizer (Stir-Pak^®^, Brinton, IL, USA) at 4 °C. Next, 100 µL of the sample (plasma or homogenized tissue) was mixed with 200 µL of trichloroacetic acid (TCA; 10%) for 30 min on ice. The mixture was subsequently centrifuged at 4500 rpm for 15 min at 4 °C, and the supernatant (200 µL) was mixed with 200 µL of thiobarbituric acid (TBA; 0.67%) and incubated in a water bath (100 °C) for 1 h. The absorbance was measured with a spectrophotometer (Thermo Electron Corporation^®^, Madison, WI, USA) at 532 nm. To ensure the reliability of the results, a calibration curve with MDA at known concentrations was created prior to analysis.

### 2.5. Antioxidant Activity

SOD activity was measured in liver, kidney, and brain tissues using a colorimetric kit in accordance with the manufacturer’s instructions (Invitrogen^®^, EIASODC, Waltham, MA, USA). To determine the GSH content (ab239727) in the kidney, a colorimetric assay was performed (Abcam^®^, Cambridge, UK).

### 2.6. Lead Determination

The lead concentration in lyophilized biological samples (brain, blood, liver, and kidney) was determined by atomic absorption spectroscopy. Freeze-dried samples were crushed into a powder in a porcelain mortar (previously cleaned with 65% nitric acid), and 0.0001 g of sample was used for mineralization via calcination at 600 °C for 6 h in a muffle furnace (Thermolyne, 6000 Furnace, Waltham, MA, USA) under a fume hood (LabconoProtector Chemical hood, Kansas City, MO, USA). Lead was quantified via atomic absorption spectroscopy (Agilent 240 FS, Santa Clara, CA, USA). Standards and samples were measured at 217 nm.

### 2.7. Data Analysis

The data were analyzed via the R programming language. Basic descriptive statistics such as the minimum, maximum, mean, and quartiles were first calculated for each variable. The Shapiro–Wilk test and the Welch’s *t*-test were subsequently performed. For nonparametric values, the median test was used. The significance level was *p* = 0.05.

## 3. Results

### 3.1. Body Weight

The mean body weight (BW) of each group was higher at the end of the study period (day 30) than on Day 0; however, the increase in the Pb group was not significant (*p* > 0.05) (Table 1). The weights of the main organs were also measured (Table 2), and the weights of the heart, liver, and kidney had decreased in the Pb group by the end of the study period (*p* < 0.05).

### 3.2. Effect of Lead Exposure on Memory

**Figure 1:**
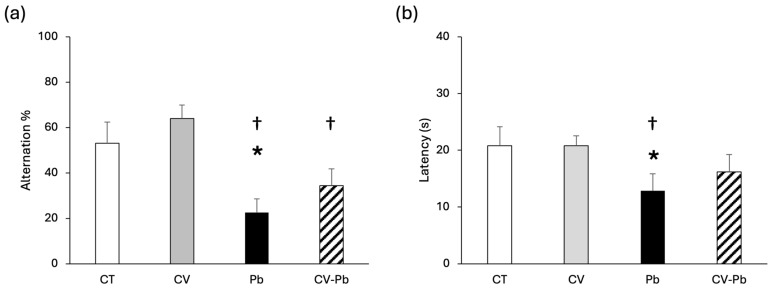
(**a**) Alteration percentage and (**b**) latency of rats in the control (CT) group, the group treated with *C. vulgaris* (CV), the group treated with lead (Pb), and the group treated with *C. vulgaris* and exposed to lead (CV–Pb). * *p* < 0.05 vs. the CT group; † *p* < 0.05 vs. the CV group.

The spontaneous alternation percentage was lower in the Pb group than in the control group (*p* < 0.05), indicating that memory was impaired by lead exposure. *C. vulgaris* supplementation restored the alternation percentage to the control level (Figure 1a). Similarly, latency decreased only in the Pb group (*p* < 0.05), and latency was restored in the group treated with *C. vulgaris* (CV–Pb) (Figure 1b).

### 3.3. Lead Concentrations in Blood and Tissue

**Figure 2:**
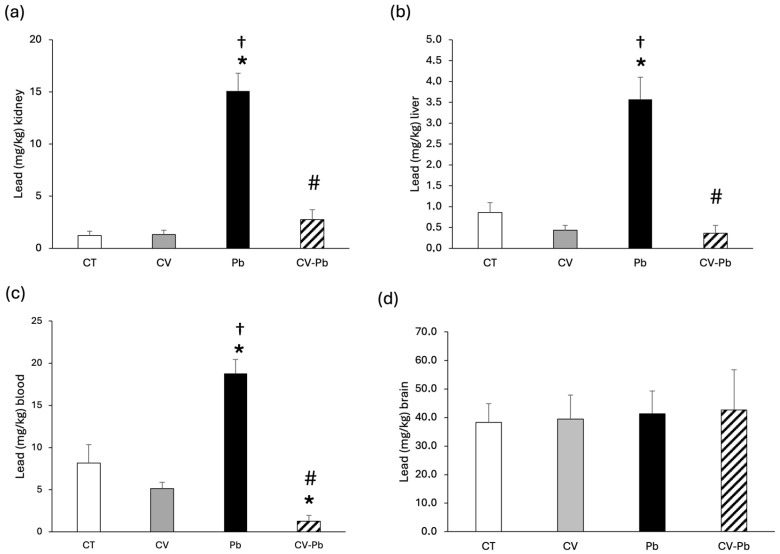
Lead concentrations in the (**a**) kidney, (**b**) liver, (**c**) blood, and (**d**) brain of rats in the control (CT) group; the group treated with *C. vulgaris* (CV); the group exposed to lead (Pb); and the group treated with *C. vulgaris* and exposed to lead (CV–Pb). * *p* < 0.05 vs. the CT group; # *p* < 0.05 for the CV–Pb group vs. the Pb group; † *p* < 0.05 vs. the CV group.

The concentration of lead in kidney and liver tissues increased in the group of rats exposed to lead (Pb; *p* < 0.05); however, supplementation with *C. vulgaris* decreased the level of lead in the CV–Pb group (*p* < 0.05) (Figure 2a,b). The level of lead in the blood also significantly increased after exposure to this heavy metal (*p* < 0.05); however, supplementation with *C. vulgaris* decreased lead in the blood to levels lower than those in the control group (*p* < 0.05) (Figure 2c). With respect to brain tissue, the concentration of lead did not differ among the study groups (*p* > 0.05) (Figure 2d).

### 3.4. Lipid Peroxidation 

**Figure 3:**
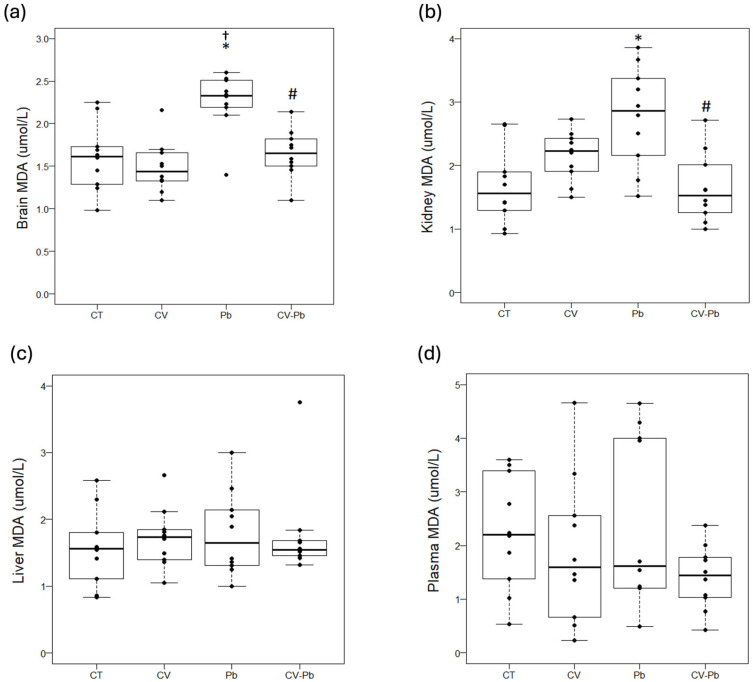
Lipid peroxidation in (**a**) brain, (**b**) kidney, (**c**) liver, and (**d**) plasma samples from rats in the control (CT) group; the group treated with *C. vulgaris* (CV); the group exposed to lead (Pb); and the group treated with *C. vulgaris* and exposed to lead (CV–Pb). * *p* < 0.05 vs. the CT group; # *p* < 0.05 for the CV–Pb group vs. the Pb group; † *p* < 0.05 vs. the CV group.

Compared with that in the control group, lipid peroxidation, as represented by the malondialdehyde (MDA) concentration, was higher in the brains of the rats exposed to lead (Pb) (*p* < 0.05). Supplementation with *C. vulgaris* in the CV–Pb group decreased the MDA level in the brains of the rats (*p* < 0.05) (Figure 3a). Similarly, the kidney MDA concentration was higher in the Pb group than in the control group (*p* < 0.05), and supplementation with *C. vulgaris* in the CV–Pb group decreased this value to the control level (*p* < 0.05) (Figure 3b). In the liver and plasma samples, the MDA concentration did not significantly differ among the study groups (*p* > 0.05) (Figure 3c,d).

### 3.5. Antioxidant Activity

**Figure 4:**
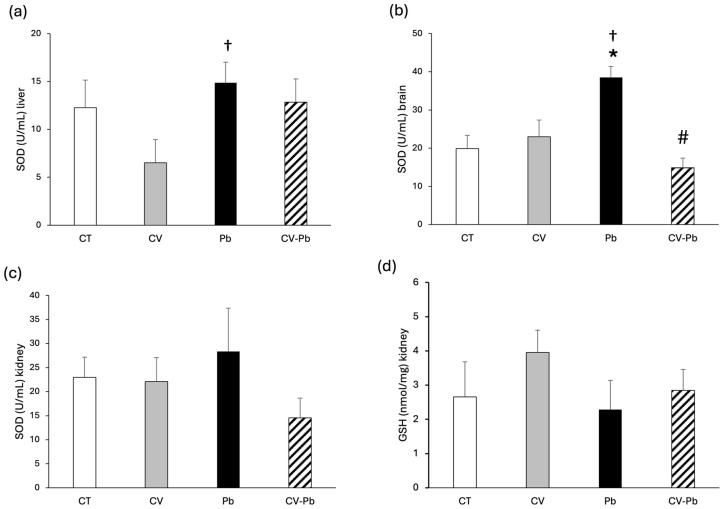
Superoxide dismutase (SOD) activity in (**a**) the liver, (**b**) brain, and (**c**) kidney, and (**d**) glutathione (GSH) content in the kidney tissue of rats in the control (CT) group; the group treated with *C. vulgaris* (CV); the group exposed to lead (Pb); and the group treated with *C. vulgaris* and exposed to lead (CV–Pb). * *p* < 0.05 vs. the CT group; # *p* < 0.05 for the CV–Pb group vs. the Pb group; † *p* < 0.05 for the Pb group vs. the CV group.

The activity of SOD in the livers of the rats in the lead group did not differ from that in the control group; however, there was a significant difference compared with that in the CV group (Figure 4a). In brain tissue, SOD activity significantly increased after exposure to lead, and supplementation with *C. vulgaris* restored SOD activity to control levels (Figure 4b). SOD activity and GSH levels in the kidney did not significantly differ among the study groups (Figure 4c,d).

## 4. Discussion

In this study, male Wistar rats exposed to lead contamination presented no significant changes in body weight gain and exhibited impaired memory function and increased concentrations of this heavy metal in the kidney, liver, and blood. With respect to oxidative stress, lead exposure increased lipid peroxidation in the kidney and brain, as well as SOD activity in the brain. Notably, *C. vulgaris* supplementation restored these altered variables to control levels.

Studies have demonstrated the health benefits and functional properties of *C*. *vulgaris*, such as its antioxidant capacity, immune system support, and detoxification effects [33]. A recent study evaluated the antioxidant properties of *C. vulgaris* and reported an antioxidant capacity of 621.83 ± 36.41 µmol/g ascorbic acid equivalent, a ferric reducing antioxidant power of 1.28 ± 0.09, a hydrogen peroxide scavenging potential of 34.04 ± 3.18% and a total phenolic content of 30.54 mg/g ellagic acid equivalent [34]. Additionally, a comparison of various extracts of *C. vulgaris* revealed that the aqueous extract presented the highest antioxidant activity, as indicated by the inhibition of scavenging (68.5%) and the highest phenolic content (3.45 mg/mL) [35].

Several studies have shown that excessive ROS are induced by heavy metal administration or contamination [36], leading to oxidative stress in animal models [37] and human studies [15]. The brain is highly susceptible to the adverse effects of oxidative stress as it has high levels of membrane polyunsaturated fatty acids, which are targets for lipid peroxidation [37].

In our study, we found significantly increased levels of MDA in the brain and kidney of rats exposed to lead acetate, a result that is consistent with the findings of other studies [38,39]. A study in male rats exposed to 500 ppm lead acetate revealed an increase in the MDA concentration and decreases in GSH and GSH-Px, with an increase in the CAT level in liver tissue; the increase in this latter antioxidant enzyme could represent the development of a defense mechanism [40]. A study on the kidneys of male rats exposed to lead acetate (0.25, 0.5, and 1.0 mg/mL) revealed increases in MDA and hydrogen peroxide (H_2_O_2_) in a dose-dependent manner [39], a finding that is consistent with our results for this organ. In terms of antioxidant activity, that study also revealed that the activities of glutathione-s-transferase, SOD, and CAT increased in a dose-dependent manner, whereas those of GSH and GSH-Px decreased [39]. The results of our study revealed no differences in SOD or GSH activity in the kidney, a finding that could be explained by the duration of the experimental procedure, which was 2 weeks shorter than the study by Oyagbemi et al. [39].

An analysis of brain antioxidants revealed a significant decrease in SOD activity after lead acetate administration [41,42] in contrast to our results, which revealed a notable increase in SOD activity after heavy metal exposure. In a study by Pande and Flora [43], 2000 ppm lead acetate was administered in drinking water, and there was no difference in SOD activity in the brain. However, studies in humans have shown that SOD activity is greater in individuals with low and medium levels of exposure to lead [15], which is concordant with our results in an animal model. Moreover, another study showed that SOD activity was increased in children with neurological disorders and higher blood lead levels [16]. Therefore, SOD activity could be associated with the concentration of lead administered.

The spatial memory evaluation in our study revealed a decrease in the spontaneous alternation percentage in the Pb group, a result that is consistent with the findings of another study in mice in which the administration of lead acetate impaired memory function [44]. However, compared with that in the control group, the percentage of alternations in the CV–Pb group was greater. The impairment in memory function could be associated with oxidative stress, as demonstrated in a previous study in rats treated with cadmium, which is another heavy metal associated with brain damage [45].

With respect to latency, we expected an increase in the Pb group; however, our results revealed a decrease in this variable. Therefore, we hypothesize that the rats make faster decisions not based on memory but rather in response to stimuli through synaptic neural alterations. This finding is corroborated by the results of a study in juvenile Sprague–Dawley rats exposed to lead; lead exposure in juvenile rats caused anxiety-like behavior through alterations in the glutamate receptor, leading to synaptic functional and morphological changes in the hippocampal CA1 pyramidal neurons [46]. This anxiety behavior has also been reported in studies involving the administration of other heavy metals such as cadmium [45]. Therefore, it is important to consider the specific behavior and context of the study. A limitation of our study is that we performed only one cognitive test; however, a different cognitive evaluation, such as Morris’s test, could support this result.

Studies in animal models have shown an increase in lead in the brain after exposure to lead acetate [47], and lead has been reported to accumulate preferentially in the parietal cortex, striatum, and thalamus [38]. However, in our study, the lead concentration in the brain did not differ among the groups studied, a finding that can be explained by the effect of the blood–brain barrier (BBB); i.e., lead acetate delays maturation in susceptible regions but does not significantly affect BBB permeability [48]. More studies are necessary to confirm these results. We did not find significant differences in brain weight, a finding that is supported by the results reported by Shalan [49] and could be explained by BBB function.

Moreover, the administration of lead acetate significantly increased lead accumulation in the blood, kidney, and liver, a finding that is corroborated by the results of other studies [50,51]; however, treatment with *C. vulgaris* significantly decreased the level of this heavy metal in those tissues, which indicates that green microalgae serve as chelating agents under lead acetate contamination conditions [52,53].

We found a decrease in organ weight (liver, kidney, and heart) in rats treated with lead acetate; however, different results concerning organ weight and heavy metal contamination have been described in the literature. Recent studies in rats administered high doses of lead (100 mg/kg) revealed significant increases in liver, kidney, and heart weights depending on the exposure time [49], an effect that could be explained by the responses to high levels of lead acetate, i.e., cell hyperplasia, apoptosis, and necrosis, and is possibly associated with lipid synthesis in these organs [54]. However, previous studies have shown a significant decrease in the absolute and relative liver weights of male rats exposed to 2 g/L lead acetate for 35 d [55] and male rats treated with 500 ppm lead for 4 weeks [40], findings that corroborate our results. A study on rats revealed a reduction in kidney weight and a general nephrotoxic effect after 4% lead acetate administration [56]. Finally, in heart studies by Li et al. [57], the heart weight index tended to decrease in a group of rats exposed to lead acetate for 14 d, but the difference was not statistically significant, possibly because of the shorter observation time. Therefore, it is important to consider the animal model used and the level of lead acetate administered as these could be important factors in organ weight during the study period.

Finally, with respect to body weight gain, previous studies have demonstrated a decrease in this variable in animals under lead exposure [58,59,60], which is concordant with our findings, which revealed nonsignificant changes in body weight gain only in the Pb group at the end of exposure (Day 30) compared with Day 0. This effect could be attributed to the alteration of central cholinergic function activation; this has been corroborated by in vivo evaluations of the acetylcholine turnover rate, which demonstrated decreases of 35% to 54% in the cortex, hippocampus, midbrain, and striatum [61,62]. Another possible explanation for these results could be related to bacteria in the intestine, as studies in mice have shown that under lead contamination, the abundance of Akkermansia microorganisms is notably reduced, affecting body weight gain [60]. Moreover, it has been reported that *C. vulgaris* can modulate the gut microbiota and protect against pathological symptoms of colitis [63], suggesting a possible explanation for the significant body weight gain in the CV–Pb group.

Taken together, these results indicate that the chelating effect of *C. vulgaris* could protect against lead accumulation, oxidative stress, and cognitive impairment; however, more studies are necessary to establish the use of *C. vulgaris* as an effective treatment or preventive agent for lead contamination in the human population.

## 5. Conclusions

Exposure to lead impacts principal organs in rats, altering their memory, an effect that could be associated with excess reactive oxygen species (ROS) or an exacerbation of oxidative stress. *C. vulgaris* supplementation had a protective effect against lead contamination, as evidenced by the restoration of memory function to the control level and a reduction in oxidative stress in brain and kidney tissues. Moreover, *C. vulgaris* exhibited important chelating effects on the blood and principal organs of rats after lead exposure. However, more studies are necessary to propose *C. vulgaris* as a powerful chelating and preventive factor in diseases associated with lead exposure in the human population.

## Figures and Tables

**Table 1:** Body weight (g) at the beginning (Day 0) and end of the experimental protocol (Day 30).

Group	BW, Day 0	BW, Day 30
CT	552.50 ± 9.41	582.30 ± 9.05 *
CV	558.15 ± 7.89	598.40 ± 9.22 *
Pb	557.20 ± 11.89	577.60 ± 12.17
CV–Pb	577.30 ± 12.18	599.50 ± 10.83 *

Abbreviations: BW: body weight; CT: control group; CV: group treated with *C. vulgaris*; CV–Pb: group treated with *C. vulgaris* plus lead; Pb: group treated with lead. Notes: * *p* < 0.05, Day 30 vs. Day 0.

**Table 2:** Organ weights (g) of rats at the end of the study period (Day 30).

Organ	CT	CV	Pb	CV–Pb
Liver	21.83 ± 0.80	22.23 ± 0.87	16.55 ± 0.61 *^†^	17.65 ± 0.72 *^†^
Kidney	4.41 ± 0.14	4.29 ± 0.14	3.87 ± 0.14 *^†^	4.14 ± 0.10
Brain	1.54 ± 0.03	1.45 ± 0.04	1.51 ± 0.09	1.49 ± 0.02
Heart	1.56 ± 0.02	1.59 ± 0.03	1.39 ± 0.05 *^†^	1.53 ± 0.03 ^#^

Abbreviations: CT: control group; CV: group treated with *C. vulgaris*; CV–Pb: group treated with *C. vulgaris* plus lead; Pb: group treated with lead. Notes: * *p* < 0.05 vs. the CT group. ^†^ *p* < 0.05 vs. the CV group. ^#^ *p* < 0.05 vs. the Pb group.

## Data Availability

Data are contained within the article.

## References

[1] Matović V., Buha A., Ðukić-Ćosić D., Bulat Z. (2015). Insight into the oxidative stress induced by lead and/or cadmium in blood, liver and kidneys. Food Chem. Toxicol..

[2] Balali-Mood M., Eizadi-Mood N., Hassanian-Moghaddam H., Etemad L., Moshiri M., Vahabzadeh M., Sadeghi M. (2025). Recent advances in the clinical management of intoxication by five heavy metals: Mercury, lead, chromium, cadmium and arsenic. Heliyon.

[3] Jedličková A., Kristeková D., Husáková Z., Coufalík P., Vrlíková L., Smutná T., Capandová M., Alexa L., Lusková D., Křůmal K. (2025). Inhaled lead nanoparticles enter the brain through the olfactory pathway and induce neurodegenerative changes resembling tauopathies. ACS Nano.

[4] Mitra P., Sharma S., Purohit P., Sharma P. (2017). Clinical and molecular aspects of lead toxicity: An update. Crit. Rev. Clin. Lab. Sci..

[5] Kumar A., Kumar A., Cabral-Pinto M.M.S., Chaturvedi A.K., Shabnam A.A., Subrahmanyam G., Mondal R., Gupta D.K., Malyan S.K., Kumar S.S. (2020). Lead toxicity: Health hazards, influence on food chain, and sustainable remediation approaches. Int. J. Environ. Res. Public Health.

[6] Ortega D.R., Esquivel D.F.G., Ayala T.B., Pineda B., Manzo S.G., Quino J.M., Mora P.C., De La Cruz V.P. (2021). Cognitive impairment induced by lead exposure during lifespan: Mechanisms of lead neurotoxicity. Toxics.

[7] Sipos P., Szentmihályi K., Fehér E., Abaza M., Szilágyi M., Blázovics A. (2003). Some effects of lead contamination on liver and gallbladder bile. Acta Biol. Szeged..

[8] Takeuchi H., Taki Y., Nouchi R., Yokoyama R., Kotozaki Y., Nakagawa S., Sekiguchi A., Iizuka K., Hanawa S., Araki T. (2021). Lead exposure is associated with functional and microstructural changes in the healthy human brain. Commun. Biol..

[9] Saleh S.R., Agwah R.G., Elblehi S.S., Ghareeb A.Z., Ghareeb D.A., Maher A.M. (2025). Combination of 10-hydroxy-decanoic acid and ZnO nanoparticles abrogates lead acetate-induced nephrotoxicity in rats: Targeting oxidative stress and inflammatory signalling. BMC Pharmacol. Toxicol..

[10] Reddy V.P. (2023). Oxidative stress in health and disease. Biomedicines.

[11] Guo X., Zheng Q., Gao W., Xiao Y., Shi L., Lin F., Xiong Y., Zhang Y., Xu Q., Wang L. (2025). Synergistic microglial modulation by laminarin-based platinum nanozymes for potential intracerebral hemorrhage therapy. Biomaterials.

[12] Zhang N., Gao M., Hu X., Wang P., Cheng Y., Wei H., Fu G., Ge J., Li H., Zhang W. (2025). Biomimetic peroxisome targets myocardial injury and promotes heart repair and regeneration. Biomaterials.

[13] Saikiran G., Mitra P., Sharma S., Kumar P.K., Sharma P. (2021). Selenium, oxidative stress and inflammatory markers in handicraft workers occupationally exposed to lead. Arch. Environ. Occup. Health.

[14] De Mello A.H., Costa A.B., Engel J.D.G., Rezin G.T. (2018). Mitochondrial dysfunction in obesity. Life Sci..

[15] Dobrakowski M., Pawlas N., Kasperczyk A., Kozłowska A., Olewińska E., Machoń-Grecka A., Kasperczyk S. (2017). Oxidative DNA damage and oxidative stress in lead-exposed workers. Hum. Exp. Toxicol..

[16] Ahamed M., Fareed M., Kumar A., Siddiqui W.A., Siddiqui M.K. (2008). Oxidative stress and neurological disorders in relation to blood lead levels in children. Redox Rep..

[17] Aaseth J., Skaug M.A., Cao Y., Andersen O. (2015). Chelation in metal intoxication—Principles and paradigms. J. Trace Elem. Med. Biol..

[18] Kim J.J., Kim Y.S., Kumar V. (2019). Heavy metal toxicity: An update of chelating therapeutic strategies. J. Trace Elem. Med. Biol..

[19] Bai Y., Ji B. (2023). Advances in responses of microalgal-bacterial symbiosis to emerging pollutants in wastewater. World J. Microbiol. Biotechnol..

[20] Panahi Y., Ghamarchehreh M.E., Beiraghdar F., Zare R., Jalalian H.R., Sahebkar A. (2012). Investigation of the effects of *Chlorella vulgaris* supplementation in patients with non-alcoholic fatty liver disease: A randomized clinical trial. Hepatogastroenterology.

[21] Barghchi H., Dehnavi Z., Nattagh-Eshtivani E., Alwaily E.R., Almulla A.F., Kareem A.K., Barati M., Ranjbar G., Mohammadzadeh A., Rahimi P. (2023). The effects of *Chlorella vulgaris* on cardiovascular risk factors: A comprehensive review on putative molecular mechanisms. Biomed. Pharmacother..

[22] Vecina J.F., Oliveira A.G., Araujo T.G., Baggio S.R., Torello C.O., Saad M.J., Queiroz M.L. (2014). Chlorella modulates insulin signaling pathway and prevents high-fat diet-induced insulin resistance in mice. Life Sci..

[23] Sugimoto Y., Taga C., Nishiga M., Fujiwara M., Konishi F., Tanaka K., Kamei C. (2002). Effect of docosahexaenoic acid-fortified *Chlorella vulgaris* strain CK22 on the radial maze performance in aged mice. Biol. Pharm. Bull..

[24] Zhang L., Tu R., Wang Y., Hu Y., Li X., Cheng X., Yin Y., Li W., Huang H. (2017). Early-life exposure to lead induces cognitive impairment in elder mice targeting SIRT1 phosphorylation and oxidative alterations. Front. Physiol..

[25] Arab H.H., Eid A.H., Alsufyani S.E., Ashour A.M., El-Sheikh A.A.K., Darwish H.W., Sabry F.M. (2023). Targeting autophagy, apoptosis, and oxidative perturbations with dapagliflozin mitigates cadmium-induced cognitive dysfunction in rats. Biomedicines.

[26] Cervantes G.I.V., Esquivel D.F.G., Ortega D.R., Ayala T.B., Chávez L.A.R., López-López H.E., Salazar A., Flores I., Pineda B., Gómez-Manzo S. (2023). Mechanisms associated with cognitive and behavioral impairment induced by arsenic exposure. Cells.

[27] Beckhauser T.F., Francis-Oliveira J., De Pasquale R. (2016). Reactive oxygen species: Physiological and physiopathological effects on synaptic plasticity. J. Exp. Neurosci..

[28] Massaad C.A., Klann E. (2011). Reactive oxygen species in the regulation of synaptic plasticity and memory. Antioxid. Redox Signal..

[29] Almeida H.N., Calixto G.Q., Chagas B.M.E., Melo D.M.A., Resende F.M., Melo M.A.F., Braga R.M. (2017). Characterization and pyrolysis of *Chlorella vulgaris* and *Arthrospira platensis*: Potential of bio-oil and chemical production by Py-GC/MS analysis. Environ. Sci. Pollut. Res. Int..

[30] Hickman D.L., Johnson S.W. (2011). Evaluation of the aesthetics of physical methods of euthanasia of anesthetized rats. J. Am. Assoc. Lab. Anim. Sci..

[31] Deacon R.M., Rawlins J.N. (2006). T-maze alternation in the rodent. Nat. Protoc..

[32] D’Isa R., Comi G., Leocani L. (2021). Apparatus design and behavioural testing protocol for the evaluation of spatial working memory in mice through the spontaneous alternation T-maze. Sci. Rep..

[33] Wang C.A., Onyeaka H., Miri T., Soltani F. (2024). *Chlorella vulgaris* as a food substitute: Applications and benefits in the food industry. J. Food Sci..

[34] Sikiru A., Arangasamy A., Comfort A., Ijaiya A., Acheneje E., Rao S.B.N. (2024). In vitro evaluation of antioxidant properties of *Chlorella vulgaris* and its derivatives for use as antioxidant supplements in animal production. Indian J. Anim. Sci..

[35] Dantas D.M.D.M., Costa R.M.P.B., Carneiro-Da-Cunha M.G., Galvez A.O., Drummond A.R., Bezerra R.S., Bezerra R.S. (2015). Bioproduction, antimicrobial and antioxidant activities of compounds from *Chlorella vulgaris*. Res. Rev. J. Bot..

[36] Balali-Mood M., Naseri K., Tahergorabi Z., Khazdair M.R., Sadeghi M. (2021). Toxic mechanisms of five heavy metals: Mercury, lead, chromium, cadmium, and arsenic. Front. Pharmacol..

[37] Khan M.H., Parvez S. (2015). Hesperidin ameliorates heavy metal induced toxicity mediated by oxidative stress in brain of Wistar rats. J. Trace Elem. Med. Biol..

[38] Villeda-Hernández J., Barroso-Moguel R., Méndez-Armenta M., Nava-Ruíz C., Huerta-Romero R., Ríos C. (2001). Enhanced brain regional lipid peroxidation in developing rats exposed to low level lead acetate. Brain Res. Bull..

[39] Oyagbemi A.A., Omobowale T.O., Akinrinde A.S., Saba A.B., Ogunpolu B.S., Daramola O. (2015). Lack of reversal of oxidative damage in renal tissues of lead acetate-treated rats. Environ. Toxicol..

[40] Ozkaya A., Sahin Z., Dag U., Ozkaraca M. (2016). Effects of naringenin on oxidative stress and histopathological changes in the liver of lead acetate administered rats. J. Biochem. Mol. Toxicol..

[41] Saxena G., Flora S.J. (2004). Lead-induced oxidative stress and hematological alterations and their response to combined administration of calcium disodium EDTA with a thiol chelator in rats. J. Biochem. Mol. Toxicol..

[42] Ferreira M.C., Zucki F., Duarte J.L., Iano F.G., Ximenes V.F., Buzalaf M.A., Oliveira R.C. (2017). Influence of iron on modulation of the antioxidant system in rat brains exposed to lead. Environ. Toxicol..

[43] Pande M., Flora S.J. (2002). Lead induced oxidative damage and its response to combined administration of alpha-lipoic acid and succimers in rats. Toxicology.

[44] Ben-Azu B., Adebayo O.G., Wopara I., Aduema W., Onyeleonu I., Umoren E.B., Kolawole T.A., Ebo O.T., Akpotu A.E., Ajibo D.N. (2022). Lead acetate induces hippocampal pyramidal neuron degeneration in mice via up-regulation of executioner caspase-3, oxido-inflammatory stress expression and decreased BDNF and cholinergic activity: Reversal effects of *Gingko biloba* supplement. J. Trace Elem. Med. Biol..

[45] Lamtai M., Chaibat J., Ouakki S., Berkiks I., Rifi E.H., Hessni A., Mesfioui A., Hbibi A., Ahyayauch H., Essamri A. (2018). Effect of chronic administration of cadmium on anxiety-like, depression-like and memory deficits in male and female rats: Possible involvement of oxidative stress mechanism. J. Behav. Brain Sci..

[46] Wang T., Guan R.L., Liu M.C., Shen X.F., Chen J.Y., Zhao M.G., Luo W.J. (2016). Lead exposure impairs hippocampus related learning and memory by altering synaptic plasticity and morphology during juvenile period. Mol. Neurobiol..

[47] Flora S.J., Saxena G., Gautam P., Kaur P., Gill K.D. (2007). Response of lead-induced oxidative stress and alterations in biogenic amines in different rat brain regions to combined administration of DMSA and MiADMSA. Chem. Biol. Interact..

[48] Moorhouse S.R., Carden S., Drewitt P.N., Eley B.P., Hargreaves R.J., Pelling D. (1988). The effect of chronic low level lead exposure on blood-brain barrier function in the developing rat. Biochem. Pharmacol..

[49] Shalan M.G. (2024). Mitigating lead acetate-induced histopathologic and physiologic disorders in rats receiving vitamin C and glutathione supplement. Heliyon.

[50] P’an A.Y., Kennedy C. (1989). Lead distribution in rats repeatedly treated with low doses of lead acetate. Environ. Res..

[51] Guimarães D., Carvalho M.L., Geraldes V., Rocha I., Alves L.C., Santos J.P. (2012). Lead in liver and kidney of exposed rats: Aging accumulation study. J. Trace Elem. Med. Biol..

[52] Queiroz M.L., Rodrigues A.P., Bincoletto C., Figueirêdo C.A., Malacrida S. (2003). Protective effects of *Chlorella vulgaris* in lead-exposed mice infected with *Listeria monocytogenes*. Int. Immunopharmacol..

[53] Sayadi M.H., Rashki O., Shahri E. (2019). Application of modified *Spirulina platensis* and *Chlorella vulgaris* powder on the adsorption of heavy metals from aqueous solutions. J. Environ. Chem. Eng..

[54] Metwally E.S.A.M., Negm F.A., El-Din R.A.S., Nabil E.M. (2015). Anatomical and histological study of the effect of lead on hepatocytes of albino rats. Int. J. Biomed. Mater. Res..

[55] Haouas Z., Sallem A., Zidi I., Hichri H., Mzali I., Mehdi M. (2014). Hepatotoxic effects of lead acetate in rats: Histopathological and cytotoxic studies. J. Cytol. Histol..

[56] Hirsch G.H. (1973). Effect of chronic lead treatment on renal function. Toxicol. Appl. Pharmacol..

[57] Li N., Zhao Y., Wang F., Song L., Qiao M., Wang T., Huang X. (2022). Folic acid alleviates lead acetate-mediated cardiotoxicity by down-regulating the expression levels of Nrf2, HO-1, GRP78, and CHOP proteins. Environ. Sci. Pollut. Res..

[58] Ibrahim N.M., Eweis E.A., El-Beltagi H.S., Abdel-Mobdy Y.E. (2012). Effect of lead acetate toxicity on experimental male albino rat. Asian Pac. J. Trop. Biomed..

[59] Mphele S.B.M., Balogun S.K., Tlhabano K.N. (2013). Effect of chronic administration of lead (Pb) on feeding behavior and weight gain among *Wistar albino* rats. IOSR J. Environ. Sci. Toxicol. Food Technol..

[60] Yang J.L., Juhasz A.L., Li M.Y., Ding J., Xue X.M., Zhou D., Ma L.Q., Li H.B. (2023). Chronic exposure to drinking water As, Pb, and Cd at provisional guideline values reduces weight gain in male mice via gut microflora alterations and intestinal inflammation. Environ. Sci. Technol..

[61] Shih T.M., Hanin I. (1978). Effects of chronic lead exposure on levels of acetylcholine and choline and on acetylcholine turnover rate in rat brain areas in vivo. Psychopharmacology.

[62] Tang M., Luo L., Zhu D., Wang M., Luo Y., Wang H., Ruan D.Y. (2009). Muscarinic cholinergic modulation of synaptic transmission and plasticity in rat hippocampus following chronic lead exposure. Naunyn Schmiedebergs Arch. Pharmacol..

[63] Velankanni P., Go S.H., Jin J.B., Park J.S., Park S., Lee S.B., Kwon H.K., Pan C.H., Cha K.H., Lee C.G. (2023). *Chlorella vulgaris* modulates gut microbiota and induces regulatory T cells to alleviate colitis in mice. Nutrients.

